# Intake of dietary advanced glycation end products influences inflammatory markers, immune phenotypes, and antiradical capacity of healthy elderly in a little‐studied population

**DOI:** 10.1002/fsn3.1389

**Published:** 2020-01-10

**Authors:** Ali Madi Almajwal, Iftikhar Alam, Mahmoud Abulmeaty, Suhail Razak, Graham Pawelec, Wajid Alam

**Affiliations:** ^1^ Clinical Nutrition Program Department of Community Health Sciences College of Applied Medical Sciences King Saud University Riyadh Saudi Arabia; ^2^ Department of Human Nutrition & Dietetics Bacha Khan University Charsadda Charsadda Pakistan; ^3^ Department of Immunology University of Tübingen Tübingen Germany; ^4^ Health Sciences North Research Institute Sudbury ON Canada; ^5^ Oral and Maxillofacial Surgery Khyber College of Dentistry Peshawar Pakistan

**Keywords:** advanced glycationed end products, aging, CRP, dAGE, immunity, inflammation

## Abstract

Dietary advanced glycation end products (dAGE) have profound negative effects on overall health, and their intake must be assessed. In this cross‐sectional study, we investigated dAGE intake of 337 adult participants (180/157:M/F; age range 50–73 years). Data were collected on anthropometrics, body composition, dietary intake, selected blood biochemistry, immunological parameters, and antiradical capacity (50% hemolysis time; HT_50_). From the dietary data, dAGEs and phytochemical index (PI) were calculated. Mean BMI, % body fat (%BF), and fasting plasma glucose were all within the accepted normal range. Subjects with high dAGE intake had higher %BF, higher energy intake, and lower PI. They tended to have lower CD4/CD8 ratios and higher proportions of B cells and NK cells, but had significantly higher hs‐CRP levels and lower HT_50_ values. Results on HT_50_ suggested that being >60 years of age enhanced dAGE‐associated impairment of defense capacity in both those with low and high HT_50_ compared with those <60 years of age. Thus, overall dAGE consumption was high, but elderly participants had lower dAGE intake than younger adults. Indicators of nutritional status and immunological parameters of the subjects were found to be associated with dAGE intake, suggesting a potential impact on health.

## Graphical abstract

dAGE intake in the daily diet of adult men was assessed in casual relation with inflammation and immune.

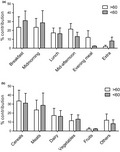

## 1 INTRODUCTION

Recently, consumption of extremely highly processed foods has greatly increased throughout the world (Cordain et al., 2005). In addition to many other alterations, this change in diet has been related to increased exposure to advanced glycation end products (AGEs), formed in foods mainly by the action of nonenzymatic browning. On the one hand, AGEs are thought to improve flavor, color, and also shelf‐life of the processed foods. On the other hand, for example, an excessive intake of AGEs can lead to a number of health disorders (Uribarri et al., 2007, 2010, 2014, 2015). AGEs are also produced inside the body during digestion, absorption, and metabolism. This is the serum/endogenous source of AGEs (sAGEs). Foods are the main exogenous source of AGEs (dietary AGEs or dAGEs) and significantly contribute to the total body's AGEs pool. The endogenous AGEs represent a minor component (Vlassara et al., 2002).

Old age is the last stage of life span. Naturally during the lifespan course, the body of an old individual has been exposed to AGEs, both dAGEs and sAGEs. AGEs may contribute to the development and severity of age‐associated diseases, including age‐associated dysregulated immunity. Many age‐associated diseases have been shown to be mediated by oxidative stress and inflammation (Uribarri et al., 2007, 2010). Accordingly, research has concentrated on the role of endogenous and exogenous AGEs in the course of the instigation and development of chronic diseases and during the aging process, signifying that AGEs may cause an elevation in the level of oxidative stress and inflammation (Uribarri et al., 2010; Vlassara et al., 2002). As an example, age‐associated elevated levels of C‐reactive protein (CRP), a commonly‐used inflammatory biomarker, have been studied as predictors of disease (Yamagishi, 2011). Associations of CRP with other disorders in old age, in particular, warrant the investigation of all dietary factors that are likely eliciting an enhanced CRP level, which may affect cellular immunity as well.

Most of the past studies on associations of AGEs with age‐associated diseases have been conducted in developed countries and, therefore, might have little utility for old people in developing countries. Less industrialized societies have little access to processed foods high in dAGEs. But at the same time, poor dietary practices common in old people in developing countries may make them more susceptible to dAGE exposure. We have recently reported on the high inflammatory potential of the diet consumed by elderly subjects in Pakistan, and we concluded that despite the seemingly balanced diet in terms of appropriate caloric content and caloric distribution in macronutrients, these diets are highly proinflammatory (Alam, Shivappa, Hebert, Pawelec, & Larbi, 2018). For the present study, we also hypothesized that the usual diets of elderly people, which are highly proinflammatory, maybe appropriate with respect to their nutritional composition but may be inappropriate with regard to higher dietary AGE contents due to inappropriate cooking methods or low quality of already‐processed food ingredients, or both. Hence, a habitual intake of such diets may cause inflammation and the related disturbance in immune parameters, importantly, T, B, and NK cells. Hence, the main objectives here were to study: (a) dAGE intake in the habitual diets of elderly people and (b) relationships between dAGE intake, inflammatory biomarkers, and immune signatures.

## 2 MATERIALS AND METHODS

### 2.1 Study site and subjects

This study was conducted in central districts of Khyber Pakhtunkhwa (KPK) of Pakistan. The participants for the present study were identified and recruited through various means. Given the scarcity of resources and other limitations, a convenience sampling design was used. The participants were approached through an organization (Nutrition Education, Awareness, and Training Organization: NEAT (Rgd), registered with the Social Welfare Department of KPK, Government of Pakistan and working for awareness and education about nutrition in the area). NEAT provided a list of individuals fulfilling the inclusion criteria. After initial selection, 450 eligible subjects were invited to participate in the study through electronic mails or phone calls. The inclusion criteria were to include healthy Pakistani subjects, nonobese, and having no recent history of infectious or noninfectious diseases. Three hundred and eighty‐one of these 450 individuals gave informed written consent documenting their willingness to participate in the study. Of these, a further 44 were ineligible and were excluded, and finally data collection could be completed on a total of 337 participants (180 men and 157 women).

### 2.2 Ethical approval, and participants' consent

The Board of Studies of Human Nutrition & Dietetics, Bacha Khan University, Pakistan, approved the design of the study and provided ethical approval (No. HN&D 21/2017). Participants were recruited through personal contacts, telephone calls, and by inviting by the already recruited participants. The study was conducted according to the criteria set by the Declaration of Helsinki, and each subject signed an informed consent before participating to the study.

### 2.3 Anthropometrics and body composition

Anthropometrics measurements were performed for all subjects as previously reported (Alam, Larbi, Larbi, Pawelec, & Paracha, 2012; Alam et al., 2018). Weight and height were measured following standard methods. Weight was measured on an electronic scale (Seca 874) to the nearest 0.1 kg. Heights were measured to the nearest 0.1 cm with a stadiometer (Seca, Model 225). Body mass index (BMI) was calculated as weight/height^2^ (kg/m^2^). Body fat was measured using the Bio‐impedance Analysis (BIA) technique using a dual‐frequency (5 kHz & 50 kHz) Bodystat ®1500MDD bioelectrical impedance analyzer (Bodystat Ltd.). Measurements were taken using a hand‐to‐foot tetra‐polar technique with participant in supine position, in accordance with the manufacturer's instruction manual.

### 2.4 Dietary data

We used 3 repeated 24‐hr dietary recalls (24‐hr) for collection of dietary data according to procedures we used for our previous studies (Alam, Larbi, et al., 2012; Alam et al., 2018). Briefly, we conducted face‐to‐face interviews with the subjects. A questionnaire with some modifications was used for three 24‐hr dietary recalls on three different days of a week at different times. During these interview sessions, the subjects were asked to recall “what they had eaten during the previous day starting from breakfast in the morning until their last meals at night before sleep” (Alam, Larbi, et al., 2012). Type of food (boiled, fried, roasted, steamed, etc) and source of food (home, hotel, others, etc) were also confirmed. In the case of old subjects, we verified the type (e.g., boiled, fried, grilled, etc) and amount of food eaten over the previous day by asking someone in the household, who usually took care of the old subject. This was done in order to avoid any over‐ or underreporting of food because old people might easily forget what exactly they had eaten over the previous 24 hr. The amount of food eaten was ascertained by asking questions in the common household measures such as cups, bowls, and spoons. All other possible measures were taken to carefully record quantities of food consumed from a particular bowl (e.g., half of the small brown bowl). We calculated average amounts of all food items over 3 days. The average amount thus calculated for each food item was used for calculation of nutrient content. Intakes were computed using an in‐house nutrient calculator (Microsoft Office Excel 2003, Microsoft Corporation). We developed this calculator for our previous studies (Alam, Larbi, et al., 2012; Alam et al., 2018) and is based on Food Composition Tables for Pakistan (Alam, Larbi, et al., 2012; Alam et al., 2018).

### 2.5 Estimation of dAGE contents and PI

We used a published database of 549 commonly consumed foods (Uribarri et al., 2007) for the estimation of dAGEs content of foods consumed by our subjects' samples of foods in this database were tested for AGEs with ELISA using a monoclonal anticarboxymethyl lysine (anti‐CML) antibody. The values for some Pakistani‐specific food items and dishes (*n* = 12) were not found in this database. We, therefore, estimated dAGE contents from similar food items/dishes in the database. For this study, the classification for dAGE intake (high or low intake) was based on the recommendations of some previous studies that assessed high and low dAGE intake in humans (Uribarri et al., 2010). Therefore, for the current study, dAGE consumption was considered high or low if it was >15,000 kU/day or <15,000 kU/day, respectively. A phytochemical index (PI) that ranks the number of calories consumed from plant‐based foods relative to overall daily calorie intake was calculated based on the method developed by McCarty (2004) {PI = [daily energy derived from phytochemical‐rich foods kJ (kcal)/total daily energy intake (kcal)] × 100}. Foods included in the phytochemical‐rich category were fruits, vegetables, legumes, whole grains, seeds, nuts, olive oil, and soy products (McCarty, 2004). Diets were established as high saturated fat diets (≥10.0% of energy), low polyunsaturated fat diets (<6.0% of energy), high cholesterol diets (≥300.0 mg/day), and high added‐sugar diets (≥10.0% of energy).

### 2.6 Clinical assessment

In clinical assessment, the subjects were evaluated for blood pressure (BP) by a registered medical practitioner with a mercury sphygmomanometer (MP4/90112, MEDEL), with the patient in the sitting position after approximately 5 min of rest in a quiet environment. Four measurements of diastolic and systolic BP were made at approximately 5‐min intervals and averaged. Subjects also reported family history of cardiovascular diseases and diabetes.

### 2.7 Collection and processing of blood samples

Blood samples (20 ml) after 8–9 hr overnight fasting were drawn from all participants from an antecubital vein. One half (10 ml) of the blood sample was used for serum separation. The other half (10 ml) was mixed with ETDA, and 5 ml of it was used for immune cells enumeration according to the protocol we previously developed (Alam, Goldeck, Larbi, & Pawelec, 2012). The remaining 5 ml was used for evaluation of total antiradical potential (Half time or HT_50_) of each individual.

#### 2.7.1 Serum separation

For serum separation, the blood sample was left for approximately 30 min to clot and then centrifuged (1500 *g*. 15 min at 4°C) within 2 hr of collection. Serum samples were aliquoted and stored in programmable freezer at −80°C until analyses.

#### 2.7.2 Biochemical analyses on serum

Total cholesterol (TC), high‐density lipoprotein cholesterol (HDL‐C), and triglycerides (TG) were measured by means of routine standard enzymatic methods using Roche Hitachi MODULAR systems. Fasting glucose was measured by the enzymatic spectrophotometric glucose oxidase–peroxidase method (GOD‐POD: Human). CRP was measured using CRP high sensitive ELISA (IBL international).

#### 2.7.3 Immunity measurement

Selected T and B lymphocytes were measured according to the protocol we previously established for frozen whole blood samples (Alam, Goldeck, et al., 2012). Briefly, the frozen venous blood samples (5 ml), obtained in EDTA treated tubes, were thawed. An aliquot (100 µl) of whole blood was taken and placed in a Wasserman tube. PE‐ or FITC‐conjugated antibody combinations (CD3, CD4, CD8, CD16, CD19, and CD56) were added to each labeled tube, mixed with a Coulter QPREP machine, and then left covered for 60 min at room temperature. Red blood cells lysis was performed, and the specimens were then stocked in the dark overnight at 4°C. On the next day, all cell suspensions were transferred to labeled Eppendorf tubes and centrifuged at 300 *g* for 5 min. After removing the supernatant resuspending the cells (10 ml PBS/1% BSA/0.01% NaAz), as a final step, 2% of paraformaldehyde was added to each tube. Lymphocyte subsets were enumerated using a flow cytometer (Coulter). Total lymphocyte count was used to count absolute number of CD3 (total T cells), CD4 (helper cells), CD8 (killer cells), CD16/CD56 (NK cells), and CD19 (B cells).

#### 2.7.4 Antiradical potential

Total antiradical potential was determined by using a biological test that is based on the principle of hemolysis induced by free radicals as reported elsewhere (Lesgards et al., 2002). Briefly, frozen blood samples were thawed and diluted to 1/50 in isotonic saline solution, to measure free radicals produced at ambient temperature and normal air conditions from the thermal decomposition of 2,2’‐azobis dihydrochloride. A 96‐well microplate reader was used for recording hemolysis measuring the optical density decay at a wavelength of 450 nm. The results were expressed as time required to reach 50% of hemolysis [half hemolysis time or HT_50_ in minutes], corresponding to resistance of whole blood to free radical.

### 2.8 Statistical analysis

Data analysis was performed using JMP (SAS). Means and standard deviations (std) were calculated. The variables of interest were analyzed according to (a) age (< or >60 years) and gender (men vs. women), and (b) according to the high dAGE intake (>15,000 kU/day) versus lower dAGE intake (<15,000 kU/day). Comparisons of baseline characteristics of participants across the age/gender levels and dAGE intake were made by using Student's unpaired *t* test (continuous variables) and chi‐square test (noncontinuous variables) or analysis of variance (ANOVA) (followed by Bonferroni correction for multiple comparisons), depending on the number of groups. Pearson correlations were performed for the dAGE intakes as continuous variables and other variables. A three‐factor multivariable analysis of variance with Wilk's lambda was used to measure the degree of correlation between the variables. This was done to measure the main effects as well as interaction effects of the independent variables (dAGE, PI and age) on HT_50,_ and hs‐CRP. A two‐tailed *p*‐value of <.05 was considered as statistically significant.

## 3 RESULTS

### 3.1 Demographics and clinical characteristics

Subjects in the current study were medically normal with no diagnosed infectious or noninfectious diseases. Except for two of the men, all subjects reported no family history of cardiovascular diseases and diabetes. Table 1 shows selected demographics, clinical, and biochemical characteristics of the subjects. The mean BMI, % BF, and fasting plasma glucose were all within the accepted normal range. Percent BF was higher in older men, although there was no age‐related change in energy intake between the two age groups of men. Compared with women, men were heavier (*p* = .03); women, as a group, had a higher % BF as compared with men, and a significant age‐related increase in % BF in men (*p* < .05) but nonsignificant in women was noted (*p* > .05). Also, men had a higher intake of calories, protein, and carbohydrates than women. In general, older age was associated with lower PI and dAGE intake; lower CD4/CD8 ratios, but higher B cells and NK cells. Similarly, older age was associated with higher systolic and diastolic blood pressure, blood cholesterol, HDL, and hs‐CRP. Older men and women had lower HT_50_ values than young men and women.

**Table 1:** General and clinical characteristics of study participants

	Men total (*n* = 180)	Men < 60 (*n* = 100)	Men > 60 (*n* = 80)	Women total (*n* = 157)	Women < 60 (*n* = 67)	Women > 60 (*n* = 80)	*p* *
Age (years)	59.4 (3.55)	53.3 (3.0)^a^	65.5 (4.1)^b^	58 (3.75)	52.9 (3.8)^c^	63.1 (3.7)^d^	.007
Weight (kg)	72.8 (16.5)	76.5 (17.2)^a^	68.9 (15.8)^b^	67.3 (18.2)	70.5 (18.4)^c^	64.1 (17.9)^d^	.001
BMI (kg/m^2^)	22.65 (2.95)	22.0 (3.1)^a^	23.3 (2.8)	21.2 (4.3)	21.7 (4.1)^c^	20.6 (4.5)^c^	.003
%BF	20.05 (5.25)	19.0 (5.8)^a^	21.1 (4.7)^b^	22.1 (5.7)	21.6 (4.1)^c^	22.5 (7.2)^c^	.098
Energy (kcal/day)	2,173.5 (351)	2,158 (336.8)^a^	2,189 (365.1)^a^	2,121.5 (417.3)	2,176 (421.8)^c^	2,067 (412.8)^d^	.2247
Protein (g/day)	59.4 (3.8)	53.3 (3.0)^a^	65.5 (4.6)^b^	56.6 (4.25)	51.8 (4.8)^c^	61.4 (3.7)^d^	.0001
Fats (g/day)	71.25 (6.6)	64.3 (12.3)^a^	78.2 (13.8)^b^	72.1 (6.55)	65.1 (19.8)^c^	79.1 (21.2)^d^	.253
PI (%)	19.75 (5.3)	21.8 (5.1)^a^	17.7 (5.5)^b^	19.2 (6.05)	21.9 (8.2)^c^	16.5 (3.9)^d^	.460
dAGE (kU/day)	17,412 (6,276.1)	21,703 (5,766.2)^a^	13,121 (6,785.1)^b^	19,618.5 (6,734.5)	24,525 (7,346.9)^c^	14,712 (6,122.8)^d^	.0027
SBP (mm Hg)	110.2 (18.25)	103.6 (15.2)^a^	116.8 (21.3)^b^	115.05 (14.75)	102.4 (16.3)^c^	127.7 (13.2)^d^	.0110
DBP (mm Hg)	71.9 (20.55)	65.2 (19.8)^a^	78.5 (21.3)^b^	77.6 (18.4)	66.9 (18.9)^c^	88.3 (17.9)^d^	.6001
Cholesterol (mg/dl)	151.7 (25.94)	145.2 (25.6)^a^	158.2 (26.3)^b^	161.3 (27.45)	151.6 (33.2)^c^	171 (21.7)^d^	.005
HDL‐C (mg/dl)	47.7 (10.5)	38.3 (9.8)^a^	57.1 (11.2)^b^	49.9 (10.6)	41.1 (11.9)^c^	58.7 (9.3)^d^	.0065
LDL‐C (mg/dl)	100.4 (21.2)	102.5 (23.4)^a^	98.3 (19.2)^b^	108.8 (28.11)	111.4 (31.2)^c^	105.3 (28.71)^d^	.0032
Triglycerides, TG (mg/dl)	95.9 (23.3)	87.6 (21.4)^a^	104.2 (25.2)^b^	105.8 (19.45)	89.4 (17.1)^c^	122.2 (21.8)^d^	.0001
Glucose (mg/dl)	87.25 (22.64)	73.2 (23.4)^a^	101.3 (21.9)^b^	91.5 (23.05)	78.3 (19.7)^c^	104.7 (26.4)^d^	.099
Hs‐CRP (mg/dl)	2.15 (1.65)	1.9 (1.6)^a^	3.9 (17)^b^	2.4 (1.6)	1.7 (1.5)^c^	3.1 (1.7)^d^	.4803
CD4:CD8	1.25 (0.55)	1.4 (0.5)^a^	1.1 (0.6)^b^	1.4 (0.75)	1.5 (0.7)^c^	1.3 (0.8)^c^	.038
B cells (% of lymphocytes)	3.6 (1.8)	3.4 (1.9)^a^	3.8 (1.7)^a^	3.9 (1.9)	3.7 (1.7)^c^	4.1 (2.1)^d^	.406
NK cells (% of lymphocytes)	2.15 (1.6)	1.9 (1.5)^a^	2.4 (1.7)^b^	1.95 (1.5)	1.8 (1.3)^c^	2.1 (1.7)^d^	.1496
HT_50_ (minutes)	128.695 (27.8)	136.29 (26.41)^a^	121.1 (29.11)^b^	97.65 (27.76)	101.5 (29.67)^c^	93.8 (25.9)^d^	.001

Note

Mean values followed by different letters as superscript (a, b) denote significant difference at *p* < .05 for men of two age categories.

Mean values followed by different letters as superscript (c, d) significant difference at *p* < .05 for women of two age categories.

Abbreviations: BF, Body fat; BMI, body mass index; dAGEs, dietary advanced glycation end products; DBP, diastolic blood pressure; HDL, high‐density lipids; Hs‐CRP, high sensitive C‐reactive protein; HT_50_, (50% hemolysis time); LDL, low density lipids; PI, phytochemical index; SBP, systolic blood pressure.

* *p*‐value calculated is two‐tailed showing difference for mean values for men total versus women total. *p* significant at <.05.

For further analysis, we divided the study subjects into two groups based on their dAGE intake (Table 2). The two groups were similar with respect to age, BMI, and protein intake, *p* > .05. However, subjects with high dAGE intake (>15,000 kU/day) had higher mean % BF, higher energy intake, and lower PI values as compared with subjects with low dAGE intake (<15,000 kU/day), *p* < .05. Diets high in dAGEs were more prevalent in subjects consuming high saturated fat diets (≥10.0% of energy), high cholesterol diets (≥300.0 mg/day), high added‐sugar diet (≥10.0% of energy), and diets with lower PI (>20%), *p* < .05. High dAGE intake was associated with significantly higher mean SBP, DBP, cholesterol, HDL‐C, triglycerides, and glucose, *p* < .05. Additionally, there was a tendency for high dAGE intake to be associated with lower CD4:CD8 ratios, higher B cells, and higher NK cells, *p* > .05. Finally, high dAGE intake was associated with higher hs‐CRP levels, *p* < .001, and with lower HT_50_ values, *p* < .001.

**Table 2:** General and clinical characteristics of the study population and dietary variables according to dAGE intake

	Low dAGE intake (<15,000 kU/day) (*n* = 196)	High dAGE intake (>15,000 kU/day) (*n* = 131)	*p* *
Age (year)	59.2 (7.6)	57.5 (7.4)	.059
BMI (kg/m^2^)	22.3 (2.19)	22.9 (2.66)	.074
BF (%)	19.3 (2.68)	23.0 (6.61)	.042
Energy (kcal)	2,013 (611)	2,307 (521)	.025
Protein (g)	69.1 (34.5)	71.2 (28.7)	.081
Fats (g)	67.9 (31.8)	78.9 (43.1)	.037
PI	21.5 (4.24)	16.2 (4.67)	.001
dAGEs (kU/day)	11,638 (6,745.3)	25,389 (6,321.8)	<.0001
High saturated fats diet (≥10.0 from energy) (%)	32.7	67.3	.002
Low polyunsaturated fats diet (<6.0% from energy) (%)	58.2	41.8	.002
High cholesterol diet (≥300.0 mg/day) (%)	28.1	71.9	.001
High added‐sugar diet (≥10.0% of energy) (%)	21.7	78.3	.002
High PI value (>20%) %	87.7	12.3	.001
SBP (mm Hg)	101 (16.8)	123.1 (18.4)	.001
DBP (mm Hg)	68.5 (14.6)	81.2 (27.3)	.001
Cholesterol (mg/dl)	151.3 (21.1)	161.5 (17.5)	.001
HDL‐C (mg/dl)	41.2 (11.8)	56.3 (12.1)	.001
LDL‐C (mg/dl)	106.2 (23.5)	102.6 (27.2)	.121
Triglycerides (mg/dl)	91.4 (22.1)	110.3 (21.8)	.001
Glucose (mg/dl)	72.5 (23.1)	106.1 (26.8)	.001
Hs‐CRP (mg/dl)	2.4 (1.32)	5.6 (1.55)	.001
CD4:CD8	1.6 (0.49)	1. 0 (0.61)	.081
B cells (% of lymphocytes)	3.4 (1.51)	3.9 (1.91)	.071
NK cells (% of lymphocytes)	1.7 (0.96)	2.7 (2.0)	.061
HT_50_ (minutes)	128.3 (27.45)	108.9 (25.8)	.001

Abbreviations: BF, body fat; BMI, body mass index; dAGE, dietary advanced glycation end product; DBP, diastolic blood pressure; HDL, high‐density lipids; Hs‐CRP, high sensitive C‐reactive protein; HT_50_, (50% hemolysis time); PI, phytochemical index; SBP, systolic blood pressure.

* *p*‐value calculated is two‐tailed. *p* significant at <.05.

### 3.2 dAGE consumption distribution in meals and food groups

**Figure 1:**
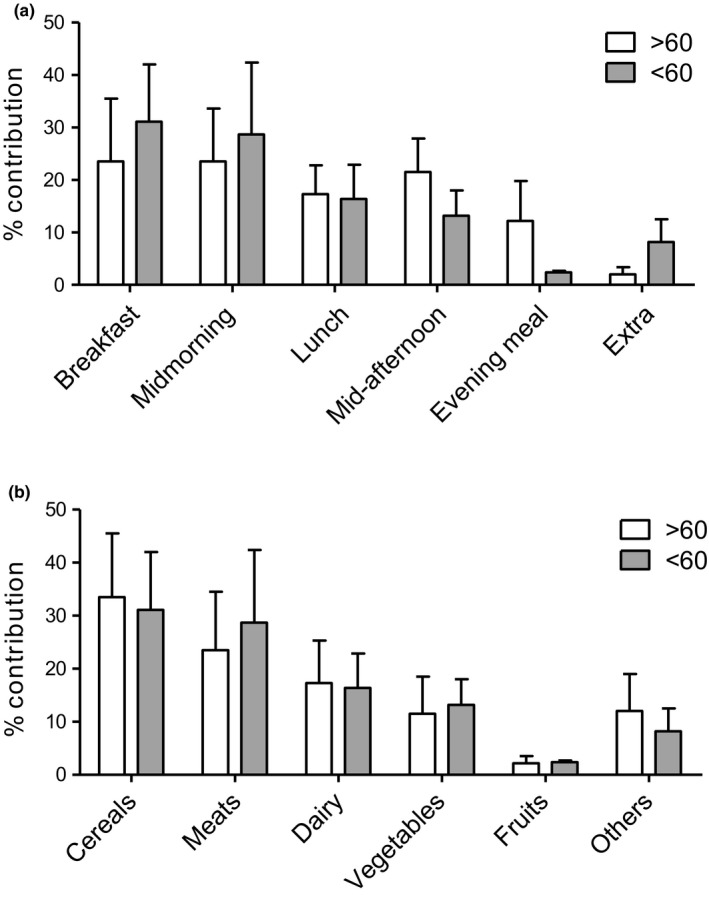
Percent dAGEs consumption distribution in meals in a day (a) and contribution of food groups in % dAGEs consumption (b). *p*‐value was calculated using two‐sample *t* test

Figure 1 shows the % dAGE consumption distribution in meals (Figure 1a). Subjects <60 year of age had the highest dAGE consumption (31.1%) from breakfast, followed by lunch (28.7%), and dinner (16.4%). Interestingly, older subjects (>60 years of age) had their highest % dAGE consumption from breakfast and midmorning meals (23.5% each), followed by midafternoon (21.5%) and evening meals (12.2%). Older subjects differed significantly from the younger subjects in % dAGE consumption from various meals. However, no significant differences in the dAGE intake patterns between men and women were observed. Figure 1b shows % contribution of food groups in dAGEs consumption. For older subjects (>60 years), cereals contributed the highest (33.5%) proportion of dAGE intake, followed by meats, dairy products and others (mostly nuts and dried fruits, etc.), and vegetables, with a % dAGE contribution of 23.5%, 17.3%, 12%, and 11.5%, respectively. Except for meat, all other food groups contributed to the same degree to dAGE intake in younger and older subjects (*p* > .05). There were no significant differences in the % contribution of various food groups in dAGE consumption between men and women.

### 3.3 Relationship of dAGE intake to hs‐CRP

dAGE intake correlated positively with serum hs‐CRP levels (*r* = .310, *p* = .041) (Table 3). The relationship remained significant after adjustment for sex, age, BMI, BF, energy intake, and PI. No independent association was found between dAGE intake and the CD4/CD8 ratio, B cells, or NK cells.

**Table 3:** Linear regression models testing the relationship of dietary AGE intake with hs‐CRP, CD4:CD8, B cells, and NK cells

	Hs‐CRP		CD4:CD8		B cells		NK cells	
	*β* (*SE*)	*p*	*β* (*SE*)	*p*	*β* (*SE*)	*p*	*β* (*SE*)	*p*
Age (years)	0.008 (0.002)	.020	0.078 (0.002)	.020	0.065 (0.092)	.010	0.072 (0.102)	.012
Gender	0.051 (0.879)	.641	0.101 (0.845)	.601	0.052 (0.879)	.641	0.081 (0.879)	.641
BMI	0.071 (0.012)	.001	0.101 (0.021)	.112	0.081 (0.012)	.101	0.073 (0.612)	.111
BF	0.062 (0.013)	.001	0.062 (0.045)	.001	0.030 (0.013)	.001	0.058 (0.042)	.041
Energy intake	0.061 (0.021)	.02	0.031 (0.054)	.051	0.051 (0.221)	.062	0.072 (0.121)	.052
dAGE intake	0.019 (0.008)	.041	0.115 (0.021)	.211	0.021 (0.008)	.211	0.082 (0.048)	.211
PI	0.018 (0.021)	.001	0.014 (0.030)	.001	0.081 (0.001)	.001	0.094 (0.011)	.011

Abbreviations: BF, body fat; BMI, body mass index; dAGE, dietary advanced glycation end product; PI, phytochemical index.

### 3.4 Combined effects of age, dAGEs, and PI on antiradical resistance (HT_50_) and hs‐CRP

**Figure 2:**
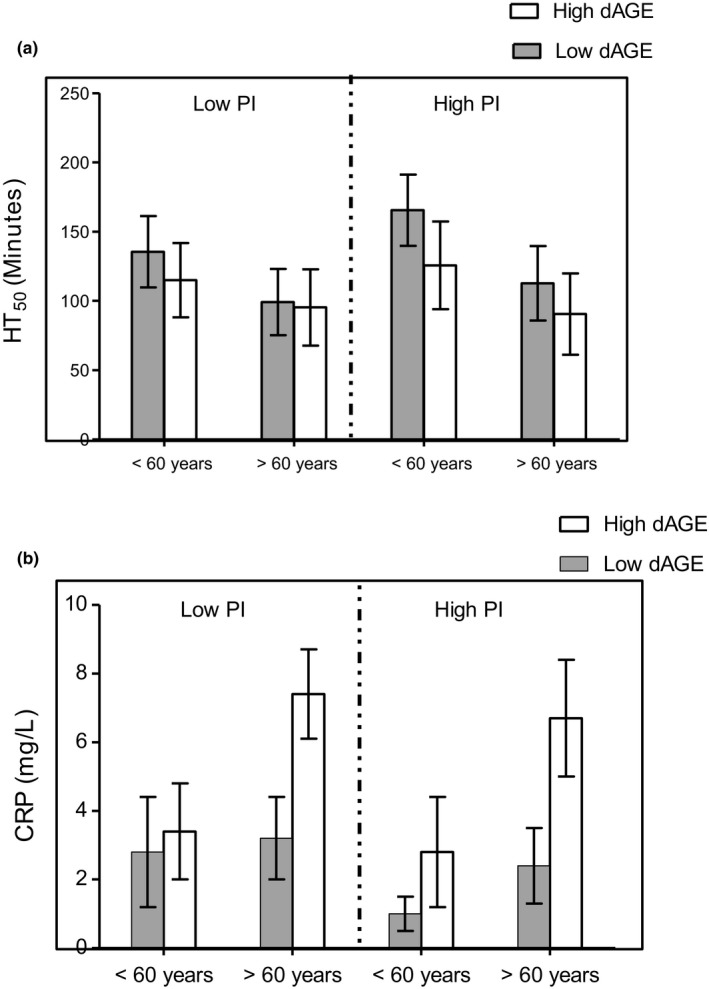
Main and combined effects of dAGE, PI, and age on HT_50_ (a) and hs‐CRP (b) of the healthy subjects. Main dAGE, PI and age effects and their interaction effects were determined with the use of a multivariate ANOVA and post hoc Bonferroni correction

We wanted to compare dAGEs and PI effects according to age of the subjects and to determine whether these effects remained independent of each other. We therefore investigated their combined effects on antiradical resistance (HT_50_) and hs‐CRP in two distinct age groups, that is, subjects <60 or >60 years of age. As depicted in Figure 2a, a 2 × 2 × 2 ANOVA showed a significant dAGE main effect (*p* < .01) as all subjects with high dAGE had lower antiradical resistance (HT_50_). Significant results were also obtained for the dAGE × PI interaction (*p* < .05) and a PI main effect (*p* < .05) as well as for an age main effect (*p* < .01). Additionally, significant results were seen for a dAGE × Age main effect (*p* < .05) and the dAGE × PI × Age interaction (*p* < .05), indicating that differences in antiradical resistance (HT_50_) between younger and older subjects depended on dAGE and PI levels of the diet.

As shown in Figure 2b, a 2 × 2 × 2 ANOVA showed a significant dAGE main effect (*p* < .01) as all subjects with high dAGE had higher hs‐CRP levels. Significant results were also obtained for PI factor, dAGE × PI, age factor, dAGE × Age main effect, and dAGE × PI × Age interaction (*p*, for all trends <.05) indicating that differences in hs‐CRP between younger and older subjects depended on dAGE and PI levels of the diet. These results broadly support the notion that both dAGE‐ and PI‐associated impairment in individual antiradical resistance (HT_50_) and inflammation (hs‐CRP) would depend on age.

## 4 DISCUSSION

In accordance with our initial hypothesis, high dAGE intake was common in the study subjects despite the fact that food was mostly prepared at home in traditional ways, and only a few cooked and/or highly processed food items or dish constituents were purchased from the market. One reason for high dAGE intake in the current study may be the use of certain food ingredients or dish constituents that are already commercially processed at high temperatures that might generate dAGEs in these food items. Examples would be commercial hydrogenated oils (local name: ghee) (Aalaei, Sjöholm, Rayner, & Tareke, 2017; Rodríguez et al., 2015), sugar (Angoorani, Ejtahed, Mirmiran, Mirzaei, & Azizi, 2016), and wheat and maize flour milled and/or processed at high temperature (Aalaei et al., 2017; Rodríguez et al., 2015). These food items are the main constituents of the staple dietary pattern of our population.

Relatively higher dAGE intake by our subjects is worrisome, bearing in mind that this intake (from a rural Pakistani sample with relatively limited access to and availability of processed food) closely resembles that of subjects from New York (city with high accessibility to, availability, and consumption of ultraprocessed foods), which have a high quantity of dAGEs (Sharma, Kaur, Thind, Singh, & Raina, 2015; Steele et al., 2016). It is difficult to establish comparisons among these studies due to differences in the study subjects and study designs; however, regional dietary patterns could explain in part these differences. While our subjects came from a rural area, where the least processed foods, and hence foods with low dAGE contents are very likely to be consumed, we assumed that subjects in urban areas and populated cities might have much higher dAGEs intake (Mendoza‐Herrera et al., 2018; Steele et al., 2016; Uribarri et al., 2014), but this seems not to be the case and therefore must be further investigated.

When we divided the subjects into two groups based on their dAGE intake, we found that those with high dAGE intake were younger, heavier, had more % BF, consumed more energy, protein, and had lower PI than their counterparts with low dAGE intake (Table 2). Compared with younger adults, elderly subjects of this study, however, had relatively low mean dAGE intakes (Table 1). These findings of low dAGE intake by the elderly are in agreement with some but not all published studies. For example, while De La Maza et al. (2007) found no differences between aged and middle‐aged participants, Uribarri et al. (2010) and Vlassara et al. (2002), separately reported decreased dAGE intake in old participants. Overall, the mean (std) dAGE intake in our study was 14,464 (5,855) kU/day [range: 4,322–25,081 kU/day], which is close to that reported in some previous studies (4,000–24,000 kU/day) (Luevano‐Contreras, Garay‐Sevilla, Wrobel, Malacara, & Wrobel, 2012; Macías‐Cervantes et al., 2015). Macías‐Cervantes et al. (2015) reported a mean intake of 14,311 kU/day in Mexican subjects (30–55 year old). Similarly, healthy New Yorkers have been reported to have dAGE intakes of 14,700 kU/day (Uribarri et al., 2007). However, our subjects had higher dAGE intakes than those reported for Iranians (9,686 kU/day) (Angoorani et al., 2016), and in another study for Mexicans (10,240 kU/day) (Mendoza‐Herrera et al., 2018).

Energy and other nutrients in the timing of daily meals in relation to diseases have recently gained considerable attention (Almoosawi, Winter, Prynne, Hardy, & Stephen, 2012). We were, therefore, tempted to investigate the dAGE consumption patterns and dietary distribution in dAGE intake. Our analysis shows considerable differences in the dAGE consumption patterns in the daily meals of adults and the elderly (Figure 1). The latter had their highest dAGE consumption contributed not by the 3 large conventional meals (i.e., breakfast, lunch, and evening meals); rather most of their dAGE intake came from snacks (midmorning and midafternoon snacks). One of the reasons for higher dAGE consumption patterns from snacks in old people might be that these meals are mostly taken outside the home and may constitute more processed (high dAGE) food items (Uribarri et al., 2015). We found no other studies that reported dAGE consumption patterns, and hence, we are unable to compare our results with others. The present results, however, may indicate poor nutritional composition of the large meals for the elderly and their dependency on snacking away from home for their nutritional requirements. There may be evident changes in temporal distribution, for example, of snacking, eating occasions, frequency of meals eaten away from home, night eating, and skipping breakfast, as reported in communities undergoing rapid socio‐economic transition. These findings may have useful implications for nutritional care plans for the elderly.

In this study, we noted higher hs‐CRP levels in subjects with high dAGE intake than in those with low dAGE intake (Table 3). Similarly, subjects with high dAGE intake had higher SBP, DBP, cholesterol, HDL‐C, triglycerides, and glucose (*p*, for all trends <.05). Similar results have been reported from other studies (e.g., Mendoza‐Herrera et al., 2018). Several other researchers have found that high dAGE intake is associated with higher concentrations of circulating AGEs, inflammatory markers, and with increased insulin resistance (Chao, Huang, Hsu, Yin, & Guo, 2010; Poulsen et al., 2013; Uribarri et al., 2007, 2014). A positive association between high dAGE intake and hs‐CRP status was found in the present study sample after adjusting for potential confounders. These results are quite similar to those of a cross‐sectional study by Uribarri et al. (2007).

Findings on the PI, in particular, are notable (Table 2). There was a small subgroup of subjects (*n* = 15 of 131; 11.5%) in the “high dAGE intake” group, who had high PI [range 21.1%–24.3%], and lower hs‐CRP levels of 0.9–1.2 mg/dl. This interesting finding may show the importance of phytochemical‐rich foods (high PI) in the diet. It seems that high intake of phytochemical‐rich foods (PI score: >20) despite high dAGE intake might have protected these individuals against the effects of dAGEs, and they did not experience an abnormal change in their hs‐CRP concentration. The concept of PI was developed in 2004 by McCarty (2004). Expressed in a score from 1 to 100, PI shows the contribution of energy from plant sources. A score >20 is usually considered an indicator of enough plant sources in the diet that could help in the maintenance of health (McCarty, 2004). On further careful analysis of their dietary habits, we found that these subjects habitually consumed higher amounts of raw green leafy vegetables added with lemon extract and/or vinegar to foods with higher dAGE contents. This might have protective effects against high dAGE intake as some foods (lemon, vinegar, and green leafy vegetables, etc) are thought to lower hs‐CRP despite higher dAGE intake (Uribarri et al., 2015). The marination of foods in low pH milieu (such as marinades with lemon or vinegar) might reduce heat‐induced formation of AGEs during cooking (Šebeková, Faist, Hofmann, Schinzel, & Heidland, 2003). However, these results need to be further explored on larger numbers of subjects with high dAGE intake and high PI values both in cross‐sectional and longitudinal studies with an intervention design. Nevertheless, these findings are very encouraging and must be considered while taking dietary data for dAGE calculations.

Subjects with high dAGE intake had lower CD4/CD8 ratios, higher B cells, and NK cells, but these differences did not reach statistical significance (Tables 2 and 3). Diets high in dAGEs activate higher levels of CRP, and hence may lead to inflammation, which may be reflected in the impaired innate immune response (Yan, Ramasamy, Naka, & Schmidt, 2003). We therefore initially hypothesized that any such dAGE‐ associated effects on hs‐CRP may be reflected in the proportion of immune cells, particularly, T lymphocytes (CD4, CD8), B, and NK lymphocytes. In the present study, CD4/CD8 ratio in subjects with high dAGE intake was lower as compared with those with low dAGE intake (1.6 vs. 1.0: Table 3). Mean % B cells and NK cells compared between subjects with higher and lower dAGE intake did not differ significantly (Table 3), although subjects with high dAGE intake had more B cells and NK cells. In short, we failed to show any significant differences between dAGE and CD4/CD8 ratio, B cells and NK cells in the present study (Table 3), even after adjustment by family history of cardiovascular disease and energy intake. There are no previous studies conducted on dAGEs and cellular immune system phenotypes, and thus, we are unable to compare our results with other studies. However, it is known that (a) AGE products may promote differentiation of CD4 lymphocytes toward proinflammatory responses (Han et al., 2014), and (b) that AGEs may interact with receptors on antigen‐presenting cells (APC), consequently, these AGEs may be presented in the context of MHC class‐II molecules to specific T lymphocytes. For example, in a mouse model, AGE‐modified ovalbumin was phagocytized quite efficiently by scavenger receptors (class‐A, types I, and II), and then expressed on myeloid dendritic cells as compared with nonmodified native OVA (Hilmenyuk et al., 2010). This enhanced antigen presentation was proposed to have led to increased activation of ovalbumin‐specific CD4 T cells (Hilmenyuk et al., 2010).

Despite lack of statistical significance, observed differences in the CD4/CD8 ratio in subjects with higher versus lower dAGEs intake are interesting results and should be further investigated for other lymphocyte subsets as well. Also, whether dAGEs, like other dietary components, for example, calories, protein, and vitamins (Alam, Goldeck, Larbi, & Pawelec, 2013; Alam, Larbi, et al., 2012; Alam, Ng, & Larbi, 2012; Alam et al., 2018; McCarty, 2004), may cause immunosenescence merits investigation. In general, the CD4/CD8 ratio may be considered as a marker for both immunosenescence and immune activation. The normal CD4/CD8 ratio in healthy hosts is clearly defined, and values 1.5–2.5 are generally considered as normal (Evans et al., 2004). A low or inverted CD4/CD8 ratio has been defined as part of an immune risk phenotype associated with mortality in elderly Swedes (Wikby et al., 2005).

While historically, inflammation was considered as a passive pathological outcome of injury or infection, at present, it is thought of as a repair and immune defense mechanism. Immune activation is usually accompanied by changes in cellular markers and other soluble factors that are associated with inflammation which is often reflected in increases in soluble biomarkers such as CRP (Alam et al., 2013). Some previous studies (Brownlee, 2001; Yan et al., 2003) have shown that dAGEs may trigger innate immunity, and this tempted us to investigate any such effect on cellular immunity. We found relatively small effects of dAGEs on the selected immune cell phenotypes, and there remains a need to adopt a more fine‐grained approach to such effects at the level of subsets of the CD4, CD8, CD19, CD56, and NK cells.

We observed a small subgroup of subjects (*n* = 15/131; 11.5%) in the “high dAGE intake” group, with a high PI but lower hs‐CRP levels (Table S1; Figure S1). This interesting observation made us analyze the possible combined effects of dAGEs and PI. We found that, despite higher PI values in subjects having high dAGE intake, there was no antagonistic or synergistic influence on either the decrease in antiradical capacity (HT_50_ values) or inflammation (hs‐CRP values) on subjects from the two age groups (Figure 2). This may be interpreted as an indication of untoward effects of both dAGEs and PI independent of each other. The interaction of the three independent variables (dAGE × PI × Age) had significant effects on the impairment of antiradical (HT_50)_ capacity and hs‐CRP (*p*, for all trends, .05). The results also demonstrated that being >60 years of age enhanced dAGE‐associated impairment of defense capacity both in individuals with low or high dAGE levels relative to those <60 years old. Being >60 years of age also showed PI‐associated impairment of defense capacity both in individuals with low or high PI values relative to those <60 years old. Very similar results were also obtained for hs‐CRP (Figure 2). The true mechanism involving these effects needs to be investigated. However, as our data suggest that lower dAGE intake was highly and positively correlated with antioxidant capacity (higher HT_50_ values) as well as lower inflammation (lower hs‐CRP value), this suggests that blood cells of individuals with high dAGE intake may have undergone more oxidative damage caused by consumption of dietary glycation compounds probably responsible for a deregulated antioxidative homeostasis (Baynes & Thorpe, 1999; Uribarri et al., 2007). These results are interesting but need further investigation both in cross‐sectional as well as longitudinal studies in animals and humans.

The present study had some limitations: Firstly, its cross‐sectional nature; secondly, use of a database originally developed from foods of Western origin, using 24 hr dietary recalls instead of a food diary for dietary intake data; and finally, lack of data on the possible confounding effects of marital/widowhood status that may influence food choices. The method of 24‐hr helps to estimate the average diet of a population, but this method is subject to significant random measurement errors due to day‐to‐day variation of the diet as well as short‐term recall error. However, we used repeated recalls over an extended period in order to minimize these errors. Although, a Food Frequency Questionnaire (FFQ) has the advantage of being less burdensome to participants and being able to assess long‐term usual diets, we did not use it because of the lack of availability of a validated dAGE‐specific FFQ. The authenticity of the dAGE database consulted for the present work has been questioned by numerous researchers mainly for the methodology of food sample preparation, insufficient characterization of the CML‐antibody, and its cross‐reactivity with other epitopes in food matrices present in thermally treated (mainly) fat‐rich foods (Delgado‐Andrade & Fogliano, 2018; Poulsen et al., 2013). Thus, data derived from the ELISA database (as done in the present investigation) and the conclusions drawn from them should be viewed critically. Liquid chromatographic methods coupled to tandem mass spectrometry in the multiple reaction monitoring mode with stable isotope dilution or standard addition represent “a gold standard” for quantification of chemically‐defined AGE‐adducts (Assar, Moloney, Lima, Magee, & Ames, 2009; Hull, Woodside, Ames, & Cuskelly, 2012; Scheijen et al., 2016). Despite all its limitations, we used this database in our present work because it covered almost all the food items reported by our subjects, while the database developed based on liquid chromatography was limited as it could hardly cover 50% of the food items reported by subjects in our current study. In addition, as far as we know, no comprehensive study has been done that has compared the two databases on the same subjects against the same parameters of interest. We are of the view that such a study is required to clearly and objectively determine how the two databases differ from each other. Nevertheless, while reproducing these results of the current study or interpreting them in relation to health or other policy‐making issues, the weaknesses and limitations of the database under discussion must be considered. To the best of our knowledge this is one of very few studies conducted in developing countries. Also, for the first time, we report effects of dAGEs on the immune signatures as well as the potential benefits of phytochemical‐rich foods via their possible protective role against dAGEs. We were also successful in establishing a relationship between dAGE intake and total antiradical potential (Half time or HT_50_). The latter is an easy biomarker for immune‐nutritional status evaluation of patients (Alam et al., 2019) and may be studied in more detail in future studies. Future studies must also focus on subjects across different age groups to clearly identify the age group at highest risk. Additionally, there is a need to develop a specific FFQ that includes all dAGE‐rich food items and that could be used for calculation of dAGE contents from intake data. Future studies should consider serum AGEs as well, to clearly establish a relationship between dAGEs and sAGEs as already done for developed countries. This will help in understanding the contribution of dAGEs to the total body pool of AGEs. Finally, we suggest studies to estimate the dAGE contents of composite dishes and intake using duplicate methods, for example, which will help in establishing dAGE contents of mixed dishes.

## 5 CONCLUSIONS

We conclude that dAGE intake is very high in adults from a rural setting in a developing country, whereas elderly subjects had a lower dAGE intake. However, compared with certain other countries in the same geographical region (e.g., Iran) and taking into account that the participants were from a rural area, their dAGE intake level seems highly alarming. We also note that dAGE intake might have triggered higher hs‐CRP levels but not necessarily affected selected immune phenotypes. Nevertheless, lower dAGE intakes showed positive correlations with antioxidant capacity and lower inflammation, suggesting that blood cells of individuals with high dAGE intake may have undergone oxidative damage caused by consumption of dietary glycation compounds probably responsible for a deregulated antioxidative homeostasis. These results need further investigation and validation both in cross‐sectional as well as longitudinal studies.

## CONFLICT OF INTEREST

All authors declare no conflict of interest.

## ETHICAL APPROVAL

The Board of Studies of Human Nutrition & Dietetics, Bacha Khan University, Pakistan, approved the design of the study and provided ethical approval (No. HN&D 21/2017). Human and animal testing was unnecessary in this study.
