# Supplementary material for: Intake of dietary advanced glycation end products influences inflammatory markers, immune phenotypes, and antiradical capacity of healthy elderly in a little‐studied population
Source: Food Sci Nutr. 2020 Jan 10;8(2):1046–57. doi: 10.1002/fsn3.1389 (PMC7020308; doi:10.1002/fsn3.1389)
Supplement: Supplementary file 1 [file FSN3-8-1046-s001.pdf]

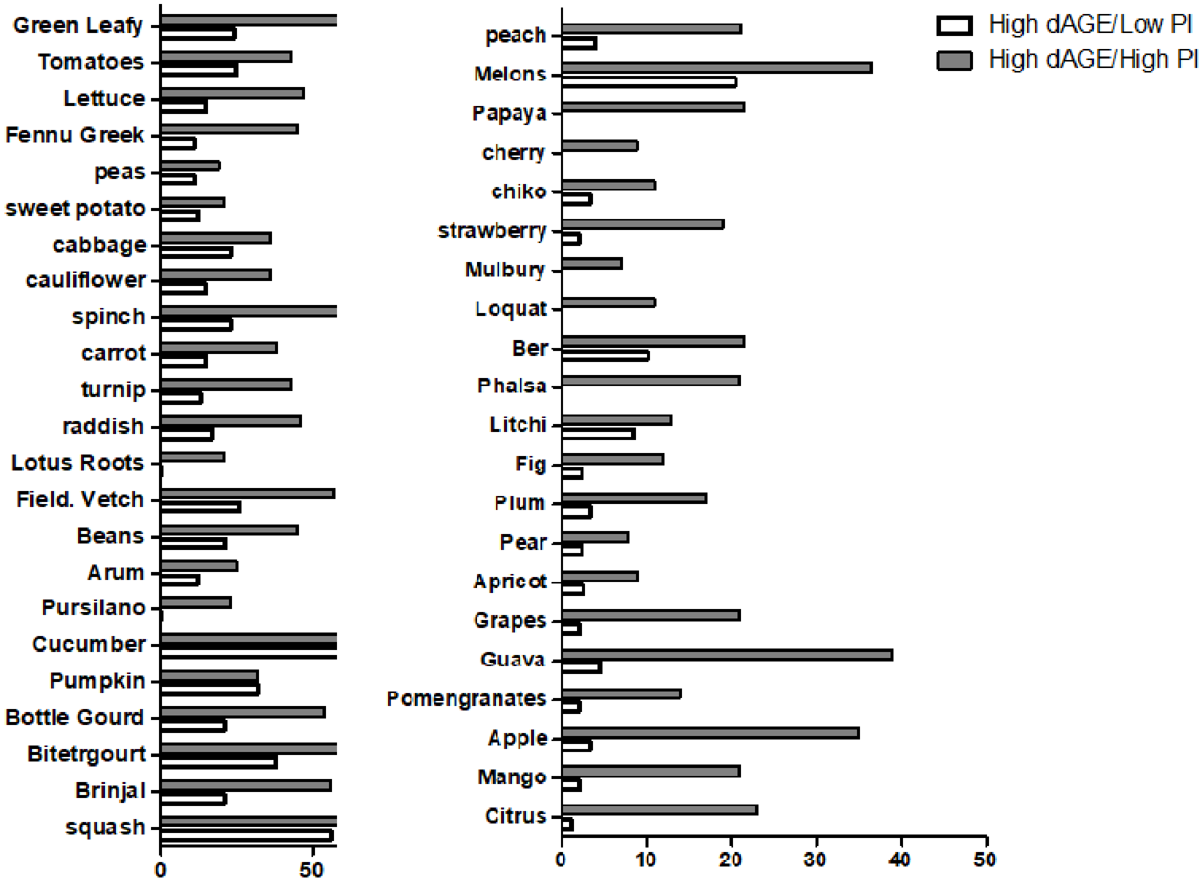

Suplementary Fig 1: Comparison between vegetables and fruits intake between the two groups with high dAGE/low PI vs. High dAGE/high PI
