# Supplementary material for: Intake of dietary advanced glycation end products influences inflammatory markers, immune phenotypes, and antiradical capacity of healthy elderly in a little‐studied population
Source: Food Sci Nutr. 2020 Jan 10;8(2):1046–57. doi: 10.1002/fsn3.1389 (PMC7020308; doi:10.1002/fsn3.1389)
Supplement: Supplementary file 2 [file FSN3-8-1046-s002.docx]

**Supplementary Table 1: Comparison of subjects with high dAGE but low PI vs. high dAGE but high PI**

|  | **High dAGE/Low PI**  **(n=196)** | **High dAGE/High PI**  **(n=15)** |
| --- | --- | --- |
| Age (yr) | 57.5 (7.4) | 58.2 (8.9) |
| BMI (Kg/m^2^) | 22.9 (2.66) | 22.4 (3.2) |
| BF (%) | 23.0 (6.61) | 22.7(5,8) |
| Energy (Kcal) | 2307 (521) | 2298 (687) |
| Protein (g) | 71.2 (28.7) | 70.3 (32.1) |
| Fats (g) | 78.9 (43.1) | 76.4 (37.2) |
| PI | 16.2 (4.67) | 22.7 (5.5)***** |
| Hs-CRP (mg/dL) | 5.6 (1.55) | 1.1 (0.6)***** |
| CD4:CD8 | 1. 0 (0.61) | 0.9 (0.41) |
| B-Cells (% of lymphocytes) | 3.9 (1.91) | 3.6 (1.61) |
| NK Cells (% of lymphocytes) | 2.7 (2.0) | 2.6 (1.41) |
| HT_50_ (minutes) | 108.9 (25.8) | 127.4 (33.5)***** |

* This symbol indicates statistically significant difference between the means at p<0.05
